# Phenotypic and genotypic profiling of swine-derived Shiga toxin-producing *Escherichia coli* over a decade in South Korea: a framework for edema disease vaccine candidate strains selection

**DOI:** 10.3389/fmicb.2025.1701708

**Published:** 2025-12-16

**Authors:** Gyeong-Seo Park, Hwan-Ju Kim, Myung A. Cho, Seung-Chai Kim, Chang-Gi Jeong, Ji-Hyun Ryu, Woo Ju Kwon, Hyo Jeong Lee, Jae-Gu Kang, Byeong Yeal Jung, Myung Hyee Kim, Chonghan Kim, Won-Il Kim, Byoung Joo Seo

**Affiliations:** 1 Vaccine Lab, WOOGENE B&G Co., LTD., Seoul, Republic of Korea; 2 College of Veterinary Medicine, Jeonbuk National University, Iksan, Republic of Korea; 3 Veterinary Diagnostic Center, Jeonbuk National University, Iksan, Republic of Korea; 4 Biosafety Research Institute, Jeonbuk National University, Iksan, Republic of Korea; 5 Korea Research Institute for Veterinary Biologics (KRIVB), Iksan, Republic of Korea

**Keywords:** Shiga toxin-producing *Escherichia coli* (STEC), porcine edema disease (ED), national surveillance, prevalence, virulence factor, vaccine candidates

## Abstract

**Introduction:**

Shiga toxin-producing *Escherichia coli* (STEC), particularly strains harboring *Stx2e*, are the primary cause of edema disease (ED) in swine, leading to acute mortality and economic loss. Despite the use of antimicrobials and autogenous vaccines, control remains inadequate due to high genetic variability and upregulated multidrug resistance (MDR). In Korea, field outbreaks persist in post-weaning pigs, underscoring the need for immunologically relevant vaccine candidates. This study aimed to characterize STEC isolates from 2014 to 2025 and identify representative strains for vaccine development through integrated molecular, phenotypic, and resistance profiling.

**Methods:**

A total of 184 STEC isolates were collected from clinical swine cases submitted between 2014 and 2025. Isolates were characterized via PCR-based virulence gene profiling, cytotoxicity assays using Vero cells, antimicrobial susceptibility testing, and Gompertz-modeled *in vitro* growth kinetics. Principal component analysis (PCA) and multivariate clustering were applied to assess phenotypic patterns. Two candidate vaccine strains were selected using an integrated scoring system incorporating virulence, cytotoxicity, metabolic fitness, and antimicrobial resistance profiles.

**Results:**

A total of 184 STEC isolates were recovered from swine diagnostic submissions across nine Korean provinces between 2014 and 2025. Genotypic analysis identified 29 virulence gene profiles, with Stx2e and F18 being predominant. Phenotypic assays revealed that Stx2e-only isolates exhibited superior growth kinetics and higher cytotoxicity than other genotypes. Antimicrobial susceptibility testing demonstrated widespread multidrug resistance, especially to sulfonamides, *β*-lactams, and aminoglycosides. Principal component analysis showed clustering based on toxin profiles and metabolic traits. No non-MDR isolates were observed, underscoring the high resistance burden. Two Stx2e-positive isolates with complementary F18 profiles, strong proliferation (>4.0 × 10^8^ CFU/mL), and consistent cytotoxicity were selected as optimal vaccine candidates. Whole-genome sequencing confirmed their genetic stability and field relevance, supporting their advancement into vaccine development pipelines.

**Conclusion:**

This study provides a decade-long surveillance-based framework for rational STEC vaccine design in swine. Two Stx2e-positive strains were selected as representative immunogen candidates based on integrated genotypic and phenotypic criteria. These findings support the development of scalable, field-relevant vaccine platforms aimed at reducing antimicrobial use and controlling edema disease in Korean pig populations.

## 1 Introduction

*Escherichia coli* (*E. coli*) is a genetically diverse Gram-negative bacterium containing multiple pathotypes, several of which are recognized as primary etiological agents of enteric diseases in livestock (Braz et al., 2020). Among these, enterotoxigenic and Shiga toxin-producing *E. coli* (ETEC and STEC, respectively) are major contributors to post-weaning diseases in pigs (Tseng et al., 2014; Kim et al., 2022). Two significant diseases, including post-weaning diarrhea (PWD) and edema disease (ED), are frequently associated with these pathotypes and lead to considerable economic losses due to mortality, reduced growth rates, and increased veterinary pharmaceutical costs. It is a major pathogen that causes post-weaning diarrhea and/or edema disease in the swine industry (Kaper et al., 2004; Tabaran and Tabaran, 2019). While PWD typically occurs as non-bloody diarrhea shortly after weaning, clinical symptoms of ED as a peracute enterotoxemic condition characterized by neurological signs, subcutaneous edema, and sudden death (Imberechts et al., 1992). In high-density swine production systems, including those in the Republic of Korea, these diseases persist despite improvements in hygiene, biosecurity, and prophylactic interventions (Heo et al., 2020).

ED is predominantly caused by STEC strains carrying the *stx2e* gene, which encodes Shiga toxin 2e (Stx2e)—an AB5-type exotoxin characterized by strong angiotropism and the ability to induce vascular endothelial damage (Imberechts et al., 1992; Seo et al., 2018; Baldo et al., 2020). Structurally, Stx2e consists of an enzymatically active A subunit that inhibits protein synthesis in target cells and five B subunits that mediate binding to specific cell surface receptors, facilitating the internalization of the A subunit and subsequent cytotoxic effects (Melton-Celsa, 2014). These strains frequently co-express F18 fimbriae, which facilitate mucosal colonization of the distal small intestine, particularly the ileum, by mediating bacterial adhesion to epithelial surfaces. Following colonization, systemic translocation of Stx2e disrupts microvascular integrity, leading to characteristic clinical signs (Berger et al., 2023). The acute progression and high fatality of ED in post-weaned piglets underscore the limited efficacy of current prevention strategies, including both vaccination and chemoprophylaxis (Gannon et al., 1989; Do et al., 2019). In many pig production settings, repetitive subtherapeutic antimicrobial exposure during the early post-weaning period remains a common treatment (Seo et al., 2018; Berger et al., 2023; Cho et al., 2023). However, this approach has contributed to the emergence and clonal expansion of multidrug-resistant (MDR) STEC, complicating therapeutic control and raising concerns regarding antimicrobial stewardship and public health risks (Holmer et al., 2019). These challenges have emphasized the necessity of effective vaccination strategies as a more sustainable alternative to antimicrobial usage.

Although autologous (farm-specific) bacterin vaccines are employed in endemic farms, their protective efficacy is often suboptimal (Sperling et al., 2022). This is largely due to the heterogeneity in adhesin types, Shiga toxin subtypes, and plasmid-encoded accessory virulence factors among field isolates (Baldo et al., 2020; Sperling et al., 2022; Lee et al., 2022). Without detailed molecular and phenotypic characterization of local strains, cross-protection remains unreliable, and vaccine performance is inconsistent across herds. Thus, a rational strain selection process, such as one anchored in both epidemiological surveillance and laboratory-based functional profiling, is critical for developing broadly protective and immunologically relevant vaccines (Eriksen et al., 2021). *In vitro* characterization offers a practical and informative platform for vaccine candidate evaluation, particularly during the preclinical phase. Targeted PCR assays allow for rapid screening of virulence-associated genes, while cytotoxicity assays using Vero cells, bacterial growth curve analyses, and quantitative adhesion tests employing porcine intestinal epithelial cell lines enable assessment of strain safety, colonization potential, and metabolic robustness (Vidotto et al., 2009; Nammuang et al., 2024; Luppi, 2017). Furthermore, determining consistent growth kinetics under laboratory conditions and verifying the stability of virulence-associated plasmids enhances candidate viability for vaccine production (Sperling et al., 2022; Matise et al., 2003). These approaches are especially valuable in early development, where animal challenge studies may be delayed or limited due to ethical, logistical, or biosafety considerations (Sperling et al., 2022).

This study aimed to investigate the prevalence and molecular diversity of STEC isolates collected from Korean pig farms between 2014 and 2025. A panel of *in vitro* assays was used to genetically characterize the strains, focusing on virulence gene profiles, cytotoxic potential, and growth properties. The ultimate goal was to identify high-virulence, genetically stable, and immunologically relevant strains as a vaccine candidate. By integrating longitudinal field surveillance with phenotypic profiling, this study establishes a framework for rational vaccine strain selection against edema disease and contributes to the broader effort to reduce STEC-associated disease burden in swine.

## 2 Materials and methods

### 2.1 Molecular epidemiology characterization of STEC isolates in swine

#### 2.1.1 Surveillance-based sample collection and regional distribution of edema disease cases in Korea (2014–2025)

From January 2014 to March 2025, clinical specimens were obtained from pigs suspected of edema disease or post-weaning diarrhea. These samples were submitted to the Jeonbuk National University Veterinary Diagnostic Center (JBNU-VDC) as part of routine veterinary diagnostic services. The submissions originated from commercial pig farms across nine provinces in the Republic of Korea and were collected without active field surveillance; therefore, sample selection was passive and case-based.

A total of 184 specimens were collected from pigs at various production stages: suckling piglets (<4 weeks of age, *n* = 12), weaning piglets (5–8 weeks, *n* = 84), growing pigs (9–16 weeks, *n* = 37), and finishing pigs (17–25 weeks, *n* = 23). For 28 cases, age information was not provided. Regional information regarding the farm of origin was recorded, and case counts were used to evaluate the spatial distribution of STEC-positive.

#### 2.1.2 Classification of clinical specimens by anatomical source

Specimens were classified based on their anatomical origin at the time of receipt. Samples included feces (*n* = 127), intestinal content or mucosal tissue (*n* = 50), and internal organ samples such as lung (*n* = 4), brain (*n* = 2), and spleen (*n* = 1). Fecal samples were typically collected from live pigs presenting with clinical signs of diarrhea. In contrast, organ tissues and intestinal samples were obtained from the dead animals submitted for postmortem examination by attending veterinarians, as part of diagnostic investigations for suspected systemic infection or enterotoxaemia. This classification was used to stratify isolation rates and investigate potential site-specific differences in STEC prevalence and virulence characteristics.

All specimens were transported in chilled containers (4–8 °C) and processed within 24 h of arrival or stored at −80 °C for delayed analysis. To maintain bacterial viability, samples were stored in a cryoprotective medium containing 25% glycerol prior to long-term preservation at −80 °C. Solid tissues were homogenized in sterile phosphate-buffered saline (PBS) prior to bacterial culture and DNA extraction. This classification was used to stratify isolation rates and investigate potential site-specific differences in STEC prevalence and virulence characteristics.

Fecal samples and homogenized tissue samples (intestine, lung, spleen, and brain) were serially diluted in phosphate-buffered saline (PBS), and 10^1^ to 10^3^ dilutions were plated onto Sorbitol MacConkey agar (SMAC, Oxoid, UK) for selective isolation of *E. coli* as previously described (Seo et al., 2018; Rivera et al., 2012). After overnight incubation at 37 °C, presumptive colonies were selected and subjected to identification using PCR targeting the virulence gene (Seo et al., 2018; Kim et al., 2010; Gao et al., 1999). Confirmed STEC isolates were routinely cultured in Luria–Bertani (LB, BD Difco™, USA) broth or Tryptic Soy Broth (TSB, BD Difco™, USA) at 37 °C with agitation. For solid culture or enumeration, the corresponding agar media (LB or TSB, respectively) were used. Mueller–Hinton agar (BD Difco™, USA) was employed for antimicrobial susceptibility testing, following established protocols (Seo et al., 2018).

### 2.2 Molecular characterization of STEC isolates based on virulence gene profiling and 16S rRNA Phylogenetics

Genomic DNA was extracted from overnight cultures of 184 *E. coli* isolates identified as Stx2e-positive Shiga toxin-producing *E. coli* (STEC), which were grown in Luria–Bertani (LB) broth or Tryptic Soy Broth (TSB) at 37 °C for 24 h. Bacterial suspensions (1 mL) were harvested by centrifugation at 10,000 × g for 5 min, and genomic DNA was extracted using the genomic DNA extraction kit (Wizard^®^ Promega, USA) according to the manufacturer’s instructions. DNA quality and quantity were assessed by spectrophotometry (A260/A280), and integrity was confirmed by agarose gel electrophoresis. The extracted DNA was used both for multiplex PCR-based detection of virulence-associated genes and for 16S rRNA gene sequencing of vaccine candidate strains.

Multiplex PCR assays targeting virulence-associated genes were performed using a commercially available kit (AccuPower^®^ Multiplex PCR PreMix, Bioneer, South Korea) in combination with specific primer sets (Table 1), as previously described (Seo et al., 2018; Zhang et al., 2007). Target genes included fimbrial adhesins (F4, F5, F6, F18, F41), enterotoxins (LT, STa, STb), and Shiga toxin 2e (Stx2e). Amplification was conducted using a thermal cycler (SimpliAmp Thermal cycler, Thermo Fisher Scientific, USA) under the following cycling conditions: initial denaturation at 95 °C for 15 min; 25 cycles of denaturation at 95 °C for 30 s, annealing at 63 °C for 90 s, and extension at 72 °C for 90 s; followed by a final extension at 72 °C for 10 min. The PCR products were held at 4 °C until analysis. Amplicons were resolved by electrophoresis on 3% agarose (BD Difco™, USA) gels containing RedSafe™ nucleic acid staining solution (iNtRON Biotechnology, South Korea) and visualized under ultraviolet light using a gel documentation system (GelDoc Go, BioRad, USA). Band sizes were compared to expected molecular weights to determine the presence of each virulence gene.

**Table 1:** Detection of virulence-associated genes from STEC isolates.

Target virulence-associated genes	Primer sequences (5′ to 3′)	PCR product size	Reference
*faeG*	Forward (F): ′TGAATGACCTGACCAATGGTGGAACC′	484 bp	Zhang et al. (2007), Seo et al. (2018)
	Reverse (R): ′GCGTTTACTCTTTGAATCTGTCCGAG′		
*fanC*	Forward (F): ′GCGACTACCAATGCTTCTGCGAATAC′	230 bp	
	Reverse (R): ′AACCAGACCAGTCAATACGAGCA′		
*fasA, fasB*	Forward (F): ′GCCAGTCTATGCCAAGTGGATACTTC′	391 bp	
	Reverse (R): ′GTTTGTATCAGGATTCCCTGTGGTGG′		
*fedA*	Forward (F): ′TGGCACTGTAGGAGATACCATTCAGC′	334 bp	
	Reverse (R): ′GGTTTGACCACCTTTCAGTTGAGCAG′		
*fim41a*	Forward (F): ′TTAGCAGCGAAGATGAGTGATGGG′	515 bp	
	Reverse (R): ′GTACTACCTGCAGAAACACCAGATCC′		
*elt*	Forward (F): ′ACGGCGTTACTATCCTGTCTATGTGC′	275 bp	
	Reverse (R): ′TTGGTCTCGGTCAGATATGTGATTCT′		
*estA*	Forward (F): ′GTCAGTCAACTGAATCACTTGACTCT′	152 bp	
	Reverse (R): ′CATGGAGCACAGGCAGGATTACAACA′		
*estB*	Forward (F): ′GCTACAAATGCCTATGCATCTACACA′	125 bp	
	Reverse (R): ′CATGCTCCAGCAGTACCATCTCTAAC′		
*stx2e*	Forward (F): ′CGGTATCCTATTCCCAGGAGTTTACG′	599 bp	
	Reverse (R): ′GTCTTCCGGCGTCATCGTATAAACAG′		

Fimbriae genes: faeG (F4), fanC (F5/K99), fasA/fasB (F6, 987P), fedA (F18), fim41a (F41); Enterotoxin genes: elt (heat-labile toxin LT), estA (heat-stable toxin STa), extB (heat-stable toxin STb); Shiga-toxin gene: stx2e.

To evaluate the molecular taxonomy of the two vaccine candidate strains selected based on virulence profile and *in vitro* characteristics, 16S rRNA gene sequencing and phylogenetic analysis were conducted. The nearly full-length 16S rRNA gene (~1.5 kb) was amplified from these two isolates using universal bacterial primers 27F (5′-AGAGTTTGATCMTGGCTCAG-3′) and 1492R (5′-GGTTACC TTGTTACGACTT-3′) as previously described (Ludwig, 2007; Al-Balawi and Morsy, 2021). Each 25-μL PCR contained 1 × PCR buffer, 0.2 mM dNTPs, 1.2 μM of each primer, 1.25 U of high-fidelity DNA polymerase (Ex Taq Hot Start, Takara, Japan), and 50–100 ng of template DNA. Thermal cycling conditions were: 95 °C for 5 min; 35 cycles of 94 °C for 1 min, 56 °C for 1 min, and 72 °C for 1 min; final extension at 72 °C for 10 min. Amplicons were verified by 1% agarose gel electrophoresis stained with ethidium bromide (0.5 μg/mL) using a 1-kb DNA ladder, purified (Expin™ PCR SV, GeneAll, Korea), and subjected to bidirectional Sanger sequencing (Cosmogenetech, Daejeon, Korea). Forward and reverse reads were assembled and quality-trimmed in SeqMan Pro (Lasergene, DNASTAR), and taxonomic identity was verified by nucleotide BLAST. For phylogeny, assembled sequences were aligned with reference Enterobacteriaceae sequences (including *E. coli*, *Shigella* spp., *Escherichia fergusonii*, and *Salmonella enterica*) using ClustalW in MEGA 11; a neighbor-joining tree was inferred with 1,000 bootstrap replicates. The resulting 16S rRNA tree is provided as Supplementary Figure S1.

### 2.3 Mammalian cell culture for cytotoxicity assay

African green monkey kidney cell line (Vero cell, ATCC^®^ CCL-81, USA) was cultured in Minimum Essential Medium *α* (MEM-*α*, Gibco™, USA) supplemented with 5% Fetal Bovine Serum (FBS, Gibco™, USA), 1% L-glutamine (Gibco™, USA), and 1% antibiotic-antimycotic cocktail (Anti-Anti, Life Technology, USA) containing 100 IU/mL penicillin, 100 μg/mL streptomycin, and 0.25 μg/mL Fungizone^®^ [amphotericin B] at 37 °C in a humidified 5% CO_2_ environment. This formulation is referred to as MEM-α growth medium.

Vero cells were maintained at 37 °C in a humidified incubator with 5% CO₂. Cells were routinely subcultured at 70–90% confluency using 0.5% trypsin–EDTA (Gibco™, USA) and then seeded into 96-well microplates 24 h prior to use in functional assays. Only cells within 10 passages from thawing were used to ensure experimental consistency. This culture system was used as a platform for performing cytotoxicity assays to distinguish STEC from various *E. coli* isolates.

#### 2.3.1 Growth kinetics and principal component analysis (PCA) of representative STEC isolates

Representative STEC isolates were cultured in Luria–Bertani (LB) broth. Overnight cultures were adjusted to an initial OD₆₀₀ of 0.05 in fresh LB and incubated at 37 °C with shaking (180 rpm). Bacterial growth was measured at 1-h intervals for 36 h using a spectrophotometer (OD₆₀₀). All experiments were performed in triplicate.

Growth curves were analyzed by nonlinear regression using the Gompertz growth model implemented in GraphPad Prism version 9.0 (GraphPad Software, USA). From the fitted curves, three kinetic parameters were derived: exponential growth rate (EGR, h^−1^), lag phase duration (LPD, h), and maximal population density (MPD, OD₆₀₀). These parameters were estimated for each isolate according to established methods (Zwietering et al., 1990; Murakami et al., 2000).

Principal component analysis (PCA) was performed using three growth-related variables—AUC, EGR, and MPD—implemented in the *FactoMineR* and *factoextra* packages in R (v4.3.0, New Zealand). The resulting PCA plots were visualized to evaluate group-level clustering patterns based on toxin profiles.

#### 2.3.2 Antimicrobial susceptibility profiling of STEC isolates by disc diffusion assay

The antimicrobial susceptibility of all *E. coli* isolates was assessed using the disc diffusion method on Mueller–Hinton agar (BD Difco™, USA), following Clinical and Laboratory Standards Institute (CLSI) guidelines (Seo et al., 2018; Zhang et al., 2007). Bacterial suspensions were standardized to a 0.5 McFarland turbidity and inoculated onto agar plates prior to application of antibiotic discs.

A total of 24 antimicrobial agents representing multiple pharmacological classes were tested (Table 2). All antibiotic discs were obtained from BD Biosciences (USA) or Thermo Scientific™ Oxoid™ (UK). Following incubation at 37 °C for 24 h, inhibition zone diameters were measured and interpreted as susceptible, intermediate, or resistant according to CLSI criteria. *Escherichia coli* (ATCC 25922) was used as the quality control strain. Isolates displaying identical susceptibility profiles across all tested agents were grouped as the same resistance type for comparative analysis. Disc diffusion was performed following CLSI-VET M100 (2023) using 24 antimicrobial agents. For clarity, resistance classification was based on inhibition zone criteria rather than MIC breakpoints; therefore, carbapenem and cephalosporin resistance results are interpreted as phenotypic screening outcomes.

**Table 2:** Antimicrobial agents used for disc diffusion assay.

No.	Antimicrobial class	Agent name	Abbreviation	Manufacturer
1	β-lactam (penicillin)	Ampicillin	Am	BD, USA
2	β-lactam (aminoglycoside)	Amikacin	An	BD, USA
3	β-lactam (carboxypenicillin)	Ticarcillin	Tic	BD, USA
4	β-lactam + *β*-lactamase inhibitor	Ticarcillin/clavulanic acid	Tim	BD, USA
5	Carbapenems	Imipenem	Ipm	Oxoid, UK
6	Cephalosporin (1st gen.)	Cephalothin	*Cf*	Oxoid, UK
7	Cephalosporin (1st gen.)	Cephalexin	CI	Oxoid, UK
8	Cephalosporin (1st gen.)	Cefazolin	Kz	Oxoid, UK
9	Cephalosporin (2nd gen.)	Cefamandole	Ma	BD, USA
10	Cephalosporin (2nd gen.)	Cefoxitin	Fox	BD, USA
11	Cephalosporin (3rd gen.)	Ceftriaxone	Cro	BD, USA
12	Cephalosporin (3rd gen.)	Ceftiofur	Ef	Oxoid, UK
13	Macrolide	Azithromycin	Azm	BD, USA
14	Aminoglycoside	Gentamicin	Gm	BD, USA
15	Aminoglycoside	Kanamycin	K	BD, USA
16	Aminoglycoside	Streptomycin	S	BD, USA
17	Quinolone	Ciprofloxacin	Cip	BD, USA
18	Quinolone (1st gen.)	Nalidixic acid	Na	BD, USA
19	Phenicol	Chloramphenicol	C	BD, USA
20	Tetracycline	Tetracycline	Te	BD, USA
21	Glycopeptide	Vancomycin	Va	BD, USA
22	Lincosamide	Lincomycin	My	Oxoid, UK
23	Sulfonamide	Sulphafurazole	Sf	Oxoid, UK
24	Sulfonamide	Sulphamethoxazole	Sx	Oxoid, UK

gen, Generation.

#### 2.3.3 Quantitative cytotoxicity profiling of Shiga toxin-producing isolates using Vero cells

Vero cells (ATCC® CCL-81™) were seeded into 96-well plates at 2 × 10^4^ cells/well and incubated overnight to reach ~80% confluency. STEC isolates were grown in Luria–Bertani broth (LB) at 37 °C with shaking (180 rpm) for 18 h. Culture supernatants were clarified by centrifugation at 3,000 × g for 20 min and sterile-filtered using 0.22 μm syringe filters.

Serial 10-fold dilutions () of filtered supernatants were prepared in MEM-*α* growth medium. A total of 180 μL of diluted supernatant was added to each well containing 20 μL of MEM-α (total volume 200 μL). Plates were incubated at 37 °C with 5% CO₂ for 24 h. Cell viability was measured using the MTT (3-(4,5-dimethylthiazol-2-yl)-2,5-Diphenyltetrazolium Bromide) assay following previously described methods in similar studies on STEC isolates (Beutin et al., 2002; da Silva et al., 2019). Briefly, 20 μL of 5 mg/mL MTT solution (Sigma-Aldrich) was added to each well, and the plates were incubated for an additional 4 h. The assay evaluates the mitochondrial reduction of tetrazolium salts into formazan crystals by metabolically active (viable) cells, providing a quantitative measure of cell viability (Murakami et al., 2000). After incubation, the medium was carefully removed, and 100 μL of dimethyl sulfoxide (DMSO) was added to dissolve the formazan crystals. The absorbance was measured at 570 nm using a microplate spectrophotometer (iMark™ Microplate Absorbance Reader, Bio-Rad Laboratories, USA).

Cytotoxicity was expressed as a percentage relative to untreated control wells. The cytotoxicity titer, representing the dilution of the supernatant that caused a reduction in cell viability of 50% or more, was calculated using non-linear regression analysis in GraphPad Prism (v9.0). The overall assay design was adapted from established methods for quantifying STEC-associated toxicity using Vero cells. The assay was applied as a functional surrogate of Stx2e-associated activity in accordance with veterinary vaccine strain screening practices; it is not toxin-subtype specific, and specificity was inferred from the consistent cytotoxicity–*stx2e* genotype correlation.

#### 2.3.4 Whole-genome sequencing of vaccine candidate STEC strains

Two representative Shiga toxin-producing *E. coli* (STEC) isolates, selected based on distinct virulence gene profiles and characteristic *in vitro* phenotypes, were subjected to whole-genome sequencing (WGS) to comprehensively elucidate their genomic properties. Genomic DNA was extracted from bacterial cultures grown overnight at 37 °C under aerobic conditions using the QIAamp PowerFecal Pro DNA Kit (Qiagen, Germany), following the manufacturer’s protocol optimized for bacterial genomic DNA. DNA quality was assessed using both a NanoDrop™ 2000 spectrophotometer (Thermo Fisher Scientific, USA) and a Qubit^®^ 4.0 fluorometer (Thermo Fisher Scientific, USA). Samples used for WGS met strict quality criteria (concentration ≥70 ng/μL, A260/A280 ratio of 1.6–2.0, and volume ≥15 μL) were selected for subsequent library preparation.

Sequencing libraries were prepared by fragmenting 100 ng genomic DNA per sample to an average insert size of approximately 350 bp using a Covaris S2 Ultrasonicator (Covaris, USA). DNA libraries were then constructed with the TruSeq Nano DNA Library Prep Kit (Illumina, USA), following the manufacturer’s guidelines. The size distribution and integrity of the prepared libraries were verified electrophoretically using an Agilent Bioanalyzer High Sensitivity DNA Kit (Agilent Technologies, USA). Library concentrations were quantified by qPCR using the KAPA Library Quantification Kit (Kapa Biosystems, USA).

Prepared libraries were subjected to cluster generation, followed by paired-end sequencing (2 × 150 bp) using the Illumina NovaSeq X Plus platform (Illumina, USA). Quality control of raw sequencing reads was performed using FastQC (v0.10.1), assessing sequence quality scores, base composition, and potential contamination. Quality-filtered reads were aligned against an appropriate reference genome using the ‘Map to Reference’ tool in Geneious Prime (v2025.2.1). Consensus sequences were generated from aligned data and subsequently annotated using Prokka (v1.14.6), enabling detailed genomic characterization.

### 2.4 Integrated evaluation for selection of field-relevant, immunologically relevant vaccine candidates

To select optimal STEC strains for *Stx2e*-based vaccine development, an integrated scoring strategy was applied to confirmed genotypic, phenotypic, and functional datasets. Candidate selection was restricted to isolates genetically verified to possess the *Stx2e* gene. Among them, candidate strains were ranked based on the following criteria:

(1) *Virulence characteristics*: Absence of adhesion-related fimbriae (e.g., F18, F41) or enterotoxin (LT, STa, STb) genes, while retaining the stx2e locus to ensure immunogenic relevance (Makino et al., 2001).
(2) *Growth fitness*: Efficient and stable *in vitro* proliferation based on Gompertz-modeled parameters (LPD, EGR, MPD) to support consistent biomass production (Zwietering et al., 1990).
(3) *Antibiotic resistance*: Inclusion of multidrug-resistant (MDR) strains exhibiting resistance to multiple antibiotic classes, including *β*-lactams and aminoglycosides, to ensure representativeness of field-circulating isolates (Nataro and Kaper, 1998; Bai et al., 2016), while maintaining biosafety considerations.
(4) *Cytotoxic potential*: High cytotoxic potential in Vero cells, expressed as quantitative cytotoxicity titers determined by MTT assays, indicative of sufficient Stx2e antigen yield for vaccine production (Zheng et al., 2008).

A weighted ranking matrix was constructed to integrate these data, enabling prioritization of field-representative and antigen-productive strains suitable for vaccine seed development. The top-ranked strains were subsequently used for immunogen formulation and preclinical evaluation.

### 2.5 Statistical analysis

All statistical analyses and data visualizations were performed using GraphPad Prism version 9.0 (GraphPad Software, USA) and R version 4.3.0, incorporating packages such as tidyverse, ggplot2, FactoMineR, and factoextra. In Prism, nonlinear regression based on the Gompertz growth model was used to calculate growth parameters, including lag phase duration (LPD), exponential growth rate (EGR), and maximum population density (MPD). Dose–response cytotoxicity data were fitted using a four-parameter logistic (4PL) model to derive cytotoxicity titers. For group comparisons involving growth kinetics, cytotoxicity, virulence gene profiles, and antimicrobial resistance rates, one-way or two-way ANOVA and Kruskal–Wallis tests were applied according to the distribution characteristics assessed by the Shapiro–Wilk normality test. *Post-hoc* analyses were conducted using Tukey’s or Dunn’s test, where appropriate. Additionally, multivariate statistical analyses were performed using R. Radar plots were generated to visualize multidrug resistance patterns across defined pathotype groups, while principal component analysis (PCA) was employed to evaluate clustering tendencies of isolates based on antimicrobial resistance traits and growth-associated parameters. Statistical differences were indicated using distinct letters or asterisks in figures (*p < 0.05,* ****p < 0.0005).

## 3 Results

### 3.1 Spatiotemporal distribution and anatomical origin of STEC isolates from Korean swine (2014–2025)

**Figure 1:**
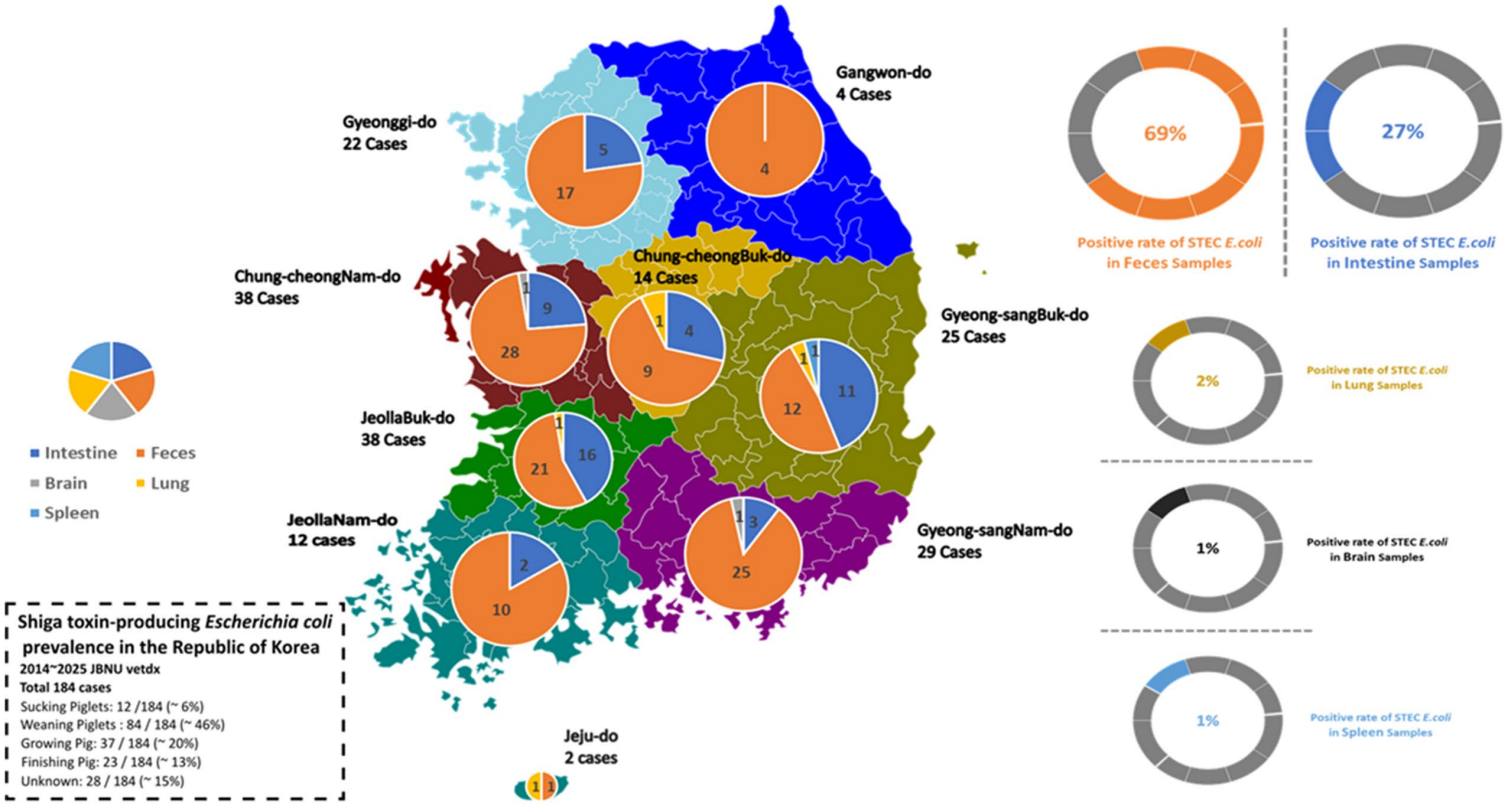
Regional and anatomical distribution of STEC isolates from Korean swine (2014–2025). A total of 184 STEC-positive cases were reported from nine provinces through diagnostic submissions to JBNU-VDC. Left: geographic case distribution by province. Right: anatomical classification, with feces identified as the most common sample type, followed by intestinal tissues. Isolates from lung, brain, and spleen were infrequent (<2% each).

A total of 184 STEC isolates were recovered from porcine diagnostic submissions to the Jeonbuk National University Veterinary Diagnostic Center (JBNU-VDC) between 2014 and 2025. These cases originated from nine administrative provinces in the Republic of Korea, with the highest number reported from JeollaBuk-do (*n* = 38), ChungcheongNam-do (*n* = 38), and GyeongsangNam-do (*n* = 29). In contrast, Jeju-do and Gangwon-do account for the lowest number of STEC-positive cases (Figure 1). Based on anatomical classification, most isolates were recovered from fecal samples (69%), followed by the intestine (27%). Isolation from systemic organs such as the lung, brain, and spleen was infrequent (< 2% each), indicating that STEC infections in swine are predominantly localized to the gastrointestinal tract (Figure 1, right panel).

**Figure 2:**
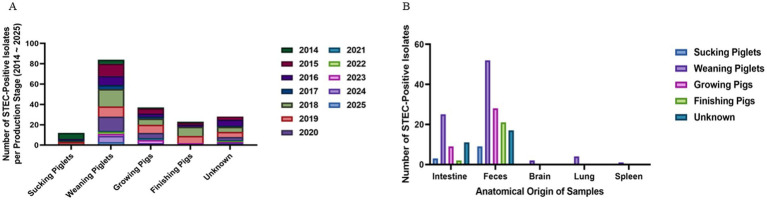
Age-related distribution and sample type of STEC isolates. **(A)** Annual distribution of STEC isolates by pig production stage. Weaning piglets (5–8 weeks) were identified for the highest number of isolates throughout the study period, followed by growing and finishing pigs. **(B)** Anatomical source distribution by production stage. Fecal samples were the predominant source in weaning piglets, while intestinal tissue was more frequently sampled from growing and finishing pigs.

When Age-based analysis observed that weaning piglets (5–8 weeks of age) comprised the most frequently affected group (84/184; 45.7%), followed by growing pigs (9–16 weeks, 37/184; 20.1%), finishing pigs (17–25 weeks, 23/184; 12.5%), and suckling piglets (<4 weeks, 12/184; 6.0%) (Figure 2A). The annual distribution of cases within each production stage remained relatively stable throughout the study period. To investigate potential differences in anatomical sampling by production stage, specimen types were further categorized by age group. Fecal samples were predominant in weaning piglets, while older pigs were more often isolated from intestinal tissues rather than feces (Figure 2B). These patterns reflect both age-specific diagnostic submission cases.

### 3.2 Genotypic characterization of virulence-associated genes and phylogenetic analysis in swine-derived STEC isolates

All 184 STEC isolates were genotyped for the presence of key virulence-associated genes, including *stx2e*, fimbrial adhesins, and enterotoxins. The most frequently detected genotype was *stx2e* alone (*n* = 44, 23.9), followed by co-detection of *stx2e* and F18 (*n* = 43, 23.4%). Additional virulence gene combinations involving STa, STb, and LT were observed in various constellations, often accompanying *stx2e* or F18. For example, isolates containing *stx2e*, F18, and both STa and STb were identified in 1.6% (*n* = 3) of the cases. Notably, 29 distinct virulence gene combinations were identified (Table 3), reflecting substantial heterogeneity among field isolates. Enterotoxins (LT, STa, STb) were more commonly associated with *F18*-positive strains than with *stx2e*-only isolates. Fimbrial variants such as F4, F5, and F41 were also sporadically detected in combination with *stx2e* or F18.

**Table 3:** Distribution of virulence gene combinations among swine-derived STEC isolates (*n* = 184) identified between 2014 and 2025.

No.	Virulence gene combination (toxin + fimbriae ± enterotoxin)	Number of isolates	Proportion (%)
1	*Stx2e*	44	24
2	*Stx2e*, F18	43	23
3	*Stx2e*, LT	2	1
4	*Stx2e*, LT, STb	1	0.5
5	*Stx2e*, STa	3	2
6	*Stx2e*, STb	4	2
7	*Stx2e*, STa, STb	4	2
8	*Stx2e*, F18, K99	1	0.5
9	*Stx2e*, F41, K99	1	0.5
10	*Stx2e*, F18, LT	3	2
11	*Stx2e*, F18, STa	4	2
12	*Stx2e*, F18, STb	3	2
13	*Stx2e*, F18, STa, STb	3	2
14	*Stx2e*, F18, LT, STa	3	2
15	*Stx2e*, F18, LT, STb	4	2
16	*Stx2e*, F18, LT, STa, STb	1	0.5
17	F18	8	4
18	F18, F41	2	1
19	F18, F41, K88, STa	1	0.5
20	F18, LT	2	1
21	F18, LT, STb	2	1
22	F18, STa	5	3
23	F18, STa, STb	25	14
24	F18, STb	9	5
25	F18, LT, STa, STb	1	0.5
26	F18, LT, STb	1	0.5
27	F18, STa, STb	2	1
28	F18, F41, LT, STa	1	0.5
29	F18, K88	1	0.5
Total		184	100.0%

Toxin gene profiles: stx2e, elt, estA, estb.

Fimbrial adhesins (protein/serotype): F18, F4, F41, K88, K99.

Combinations represent the presence of toxin genes and fimbrial adhesions detected in each isolate.

The 16S rRNA analysis confirmed that both isolates (23–0932 and 24–0997) belong to the genus *Escherichia*. Strain 23–0932 showed close clustering with *Shigella* spp., while strain 24–0997 appeared near *Salmonella enterica* Ty2 within the *Enterobacteriaceae* clade. This pattern reflects the high 16S sequence similarity among these genera rather than taxonomic misclassification. Both strains exhibited >99% sequence identity with *E. coli* references, supporting their identification as *E. coli* (Supplementary Figure 1).

### 3.3 Functional characterization of representative STEC isolates based on phenotypic analysis

#### 3.3.1 Comparative growth kinetics analysis of *Stx2e*-positive and *Stx2e*-negative isolates

To evaluate the *in vitro* growth characteristics of STEC isolates categorized by toxin gene profiles, we conducted a comparative kinetic analysis among three groups: Stx2e-only, Stx2e + fimbriae/enterotoxin, and non-Stx2e. Growth was monitored over 36 h at an optical density of 600 nm (OD₆₀₀), and corresponding area under the curve (AUC) and principal component analyses (PCA) were performed based on the growth parameters.

**Figure 3:**
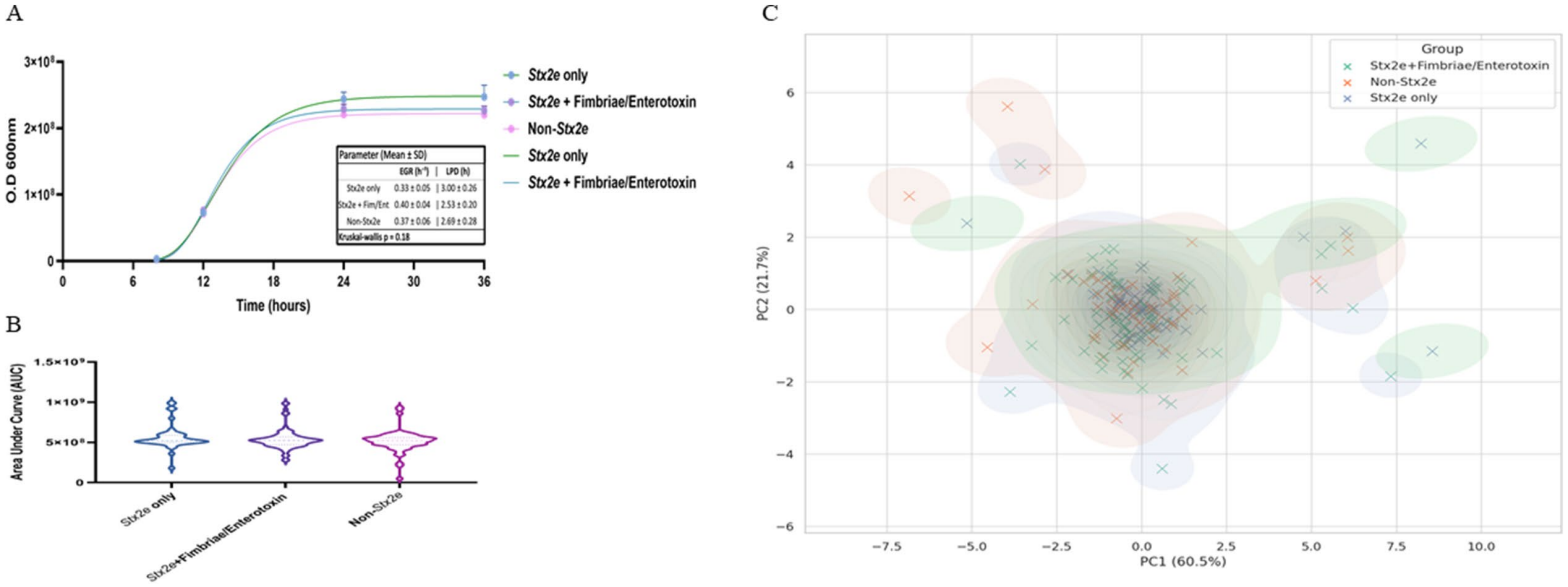
*In vitro* growth dynamics and multivariate clustering of STEC isolates classified by toxin gene profile. **(A)** Representative growth curves of STEC isolates grouped as Stx2e-only, Stx2e + fimbriae/enterotoxin, and non-Stx2e, cultured in LB broth at 37 °C with shaking. Optical density (OD_600_) was measured over 36 h. Data points represent mean ± standard deviation (SD). Solid lines depict nonlinear regression fitting using the Gompertz growth model. Representative Gompertz growth curves of three STEC pathotypes. Mean ± SD growth parameters: EGR (h^−1^): Stx2e only 0.33 ± 0.05; Stx2e + Fim/Ent 0.40 ± 0.04; Non-Stx2e 0.37 ± 0.06; LPD (h): 3.00 ± 0.26, 2.53 ± 0.20, 2.69 ± 0.28, respectively (*p* = 0.18). Error bars represent mean ± SD from triplicate cultures. **(B)** Violin plots representing the distribution of area under the curve (AUC) values derived from growth kinetics for each pathotype group. Median values are indicated by central horizontal lines. Although no statistically significant differences were detected, the Stx2e-only group exhibited the highest median AUC. **(C)** Principal component analysis (PCA) of isolates based on three growth-related variables: AUC, maximal OD_600_, and calculated growth rate. The first and second principal components explain 60.5% and 21.7% of the variance, respectively. Each point represents an individual isolate, colored by group. Kernel density contours illustrate the overall distribution tendency of each group, showing partial clustering with overlapping patterns. Statistical analysis was performed using the Kruskal–Wallis test followed by Dunn’s multiple comparison correction (*n* = 3 biological replicates per group).

Growth curve analysis demonstrated typical sigmoid proliferation patterns in all three groups of STEC isolates. Across the early exponential phase (6–12 h), isolates classified as Stx2e-only showed a more rapid increase in optical density (OD₆₀₀) compared to the other groups. This trend was sustained through the stationary phase (post-12 h), where the Stx2e-only group reached a slightly higher maximal OD. The Stx2e + fimbriae/enterotoxin group exhibited a comparable growth trajectory but with slightly lower stationary phase, whereas non-*Stx2e* isolates demonstrated a delayed transition to exponential growth and a lower overall OD value. These findings suggest that the presence of Stx2e, particularly in the absence of additional colonization factors, may confer a marginal growth advantage under standardized in vitro conditions, possibly reflecting differences in metabolic burden or regulatory interactions associated with virulence gene expression (Figure 3A). The *Gompertz* model fitting (*R*^2^ = 0.71–0.83) revealed comparable overall growth kinetics among all pathotype groups. The *Stx2e* + fimbriae/enterotoxin group showed the highest estimated specific growth rate (EGR, *K* = 0.396 ± 0.040 h^−1^; 95% CI 0.270–0.406) and the shortest lag-phase duration (1/*K* = 2.53 ± 0.20 h). The Stx2e-only and non-Stx2e groups exhibited similar parameters (*K* = 0.334 ± 0.048 and 0.373 ± 0.057 h^−1^; 1/*K* = 3.00 ± 0.26 and 2.69 ± 0.28 h, respectively). No significant difference was observed among the groups (Kruskal–Wallis *p* = 0.18), consistent with the overlapping AUC distributions (Figure 3B).

Principal component analysis (PCA) was performed using three variables: area under the curve (AUC), maximal optical density (OD₆₀₀), and estimated growth rate. The first two principal components (PC1 and PC2) explained 60.5 and 21.7% of the total variance, respectively. Distribution of isolates on the PCA plot showed partial group-wise clustering based on toxin profile. Most Stx2e-only isolates were distributed in the upper-right quadrant, while non-*Stx2e* isolates were spread across the lower-left and central regions. The Stx2e + fimbriae/enterotoxin group exhibited a broader distribution, overlapping with both other groups. No distinct separation among groups was observed, although the overall density contours showed differences in centroid positions and dispersion patterns (Figure 3C).

#### 3.3.2 Antimicrobial resistance patterns of selected *Stx2e*-positive/negative isolates

**Figure 4:**
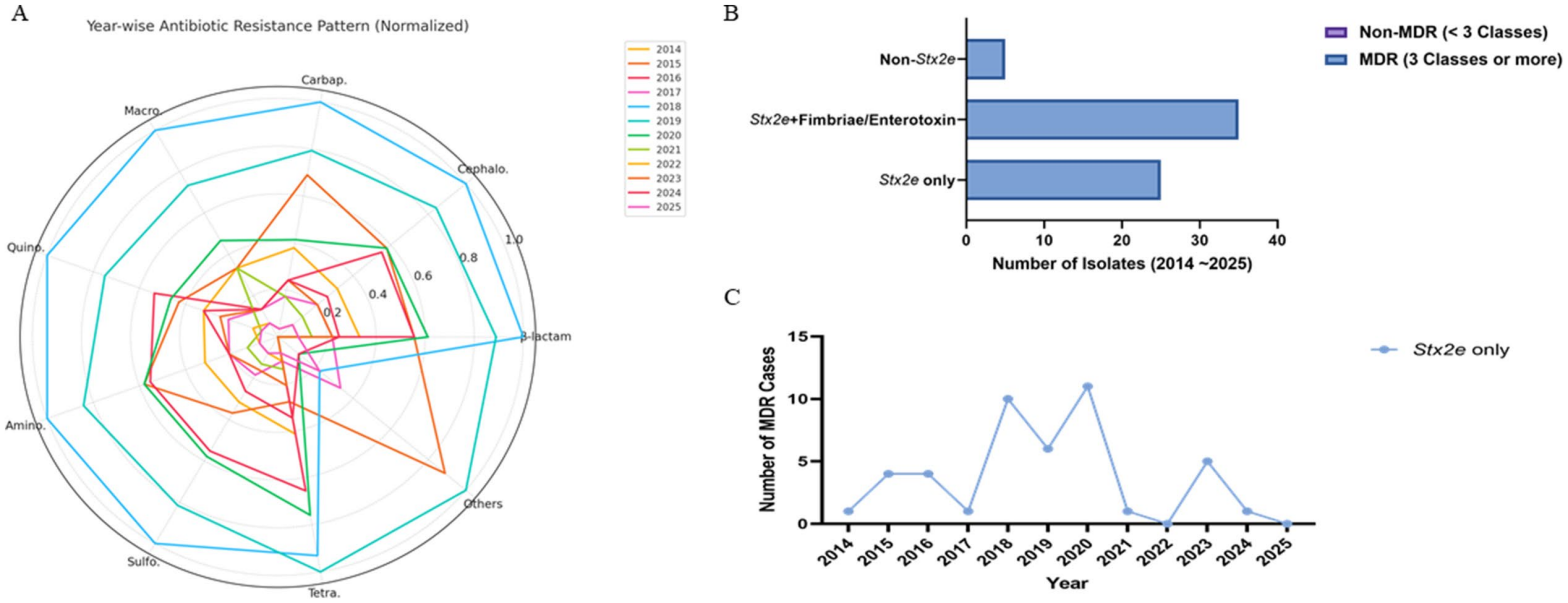
Antimicrobial resistance patterns of STEC isolates stratified by toxin gene profile and sampling year. **(A)** Radar plot visualizing the normalized distribution of detected antibiotic resistance classes from 2014 to 2025. Each axis represents a distinct antimicrobial class, and year-specific resistance levels are plotted as scaled values (0–1) relative to the highest observed class-level frequency. Yearly denominators (*n* = 12, 15, 19, 21, 24, 23, 18, 17, 14, 13, 8, 5) are indicated in parentheses for each year. A consistent presence of B-lactam, cephalosporin, and sulfonamide resistance was observed across years, with peaks in 2018–2020, driven primarily by isolates harboring Stx2e. **(B)** Stacked bar chart showing MDR (23 classes) and non-MDR (<3 classes) counts per group. Non-MDR counts were zero in both Stx2e-positive groups; the non-Stx2e group included predominantly non-MDR isolates (numerical labels shown on bars). **(C)** Yearwise trend in the number of MDR isolates identified within the Stx2e only group. While temporal fluctuation was observed, peak MDR occurrence was noted between 2018 and 2020, followed by a gradual decline. Data are presented as descriptive, exploratory visualizations; statistical comparisons were not adjusted for multiple testing, consistent with field-level AMR trend analysis in veterinary surveillance. Data represent descriptive, exploratory visualizations; statistical comparisons were not adjusted for multiple testing.

Antimicrobial resistance profiles were assessed across 184 STEC isolates collected from 2014 to 2025. A total of nine major antibiotic classes were considered, including *β*-lactams, cephalosporins, carbapenems, macrolides, quinolones, aminoglycosides, sulfonamides, tetracyclines, and others (glycopeptides and lincosamides). Among these, sulfonamides (*n* = 183), cephalosporins (*n* = 184), and aminoglycosides (*n* = 182) were the most frequently represented classes, followed by *β*-lactams (*n* = 177), tetracyclines (*n* = 155), and carbapenems (*n* = 126). Macrolide and quinolone resistance were observed in 53 and 127 cases, respectively. The cumulative resistance burden demonstrated a peak in 2019–2020, particularly for β-lactams, sulfonamides, and cephalosporins. Resistance to at least five or more classes was common from 2018 through 2020 (Table 4). When visualized by year and normalized per class, β-lactam, sulfonamide, and aminoglycoside resistance were consistently dominant. Carbapenem and cephalosporin resistance remained widespread from 2015, while macrolide and quinolone resistance fluctuated year to year with an uptick during 2018–2020 (Figure 4A).

**Table 4:** Year-wise distribution of detected antibiotic resistance classes among STEC isolates (2014–2025).

Year	Classes of antibiotics								
	*β*-lactam	Cephalo.	Carbap.	Macro.	Quino.	Amino.	Sulfo.	Tetra.	Others (Glyco., Linco)
2014	12	12	11	5	9	12	12	12	0
2015	20	22	20	5	12	22	22	14	8
2016	20	21	7	2	15	21	21	19	0
2017	8	8	5	2	6	8	8	7	3
2018	36	38	29	15	28	38	38	27	2
2019	32	32	23	11	21	32	31	29	9
2020	22	22	12	7	13	22	22	22	1
2021	5	5	5	0	2	5	5	4	0
2022	2	3	1	1	3	3	3	3	0
2023	8	8	5	2	7	8	8	6	0
2024	9	10	7	2	9	8	10	10	1
2025	3	3	1	1	2	3	3	2	2
Total	177	184	126	53	127	182	183	155	26

Cepholo., cephalosporin; Carbp., carbapenems; Marco., macrolide; Quino., quinolone; Amino., aminoglycoside; Sulfo., sulfonamide; Tetra., tetracycline; Glyco., glycopeptide; Linco., lincosamide.

Multidrug resistance (MDR), defined as resistance to three or more antimicrobial classes, was predominantly observed in Stx2e-only and Stx2e + fimbriae/enterotoxin isolates. A total of 36 MDR isolates were identified in the Stx2e + fimbriae/enterotoxin group, while 23 MDR isolates were detected in the *Stx2e*-only group. In contrast, the non-Stx2e group accounted for only three MDR cases, with the remaining isolates classified as non-MDR. Among the Stx2e-positive isolates, all strains met the MDR definition (≥3 antimicrobial classes), although the degree and spectrum of resistance varied by group (Figure 4B). Temporal profiling of MDR among *Stx2e*-only isolates revealed persistent detection from 2014 to 2021. A sharp increase in MDR prevalence occurred in 2018–2020, with 10–12 MDR cases annually, followed by a decrease in MDR cases in 2022–2025 (Figure 4C). These phenotypic resistance results represent disc-diffusion screening under field-level conditions. Carbapenem and cephalosporin resistance patterns indicate reduced inhibition-zone responses rather than confirmed enzymatic resistance. Representative isolates will be subjected to MIC testing and β-lactamase/carbapenemase genotyping in follow-up validation.

#### 3.3.3 Quantitative cytotoxicity profiles assessed by Vero cell assay

**Figure 5:**
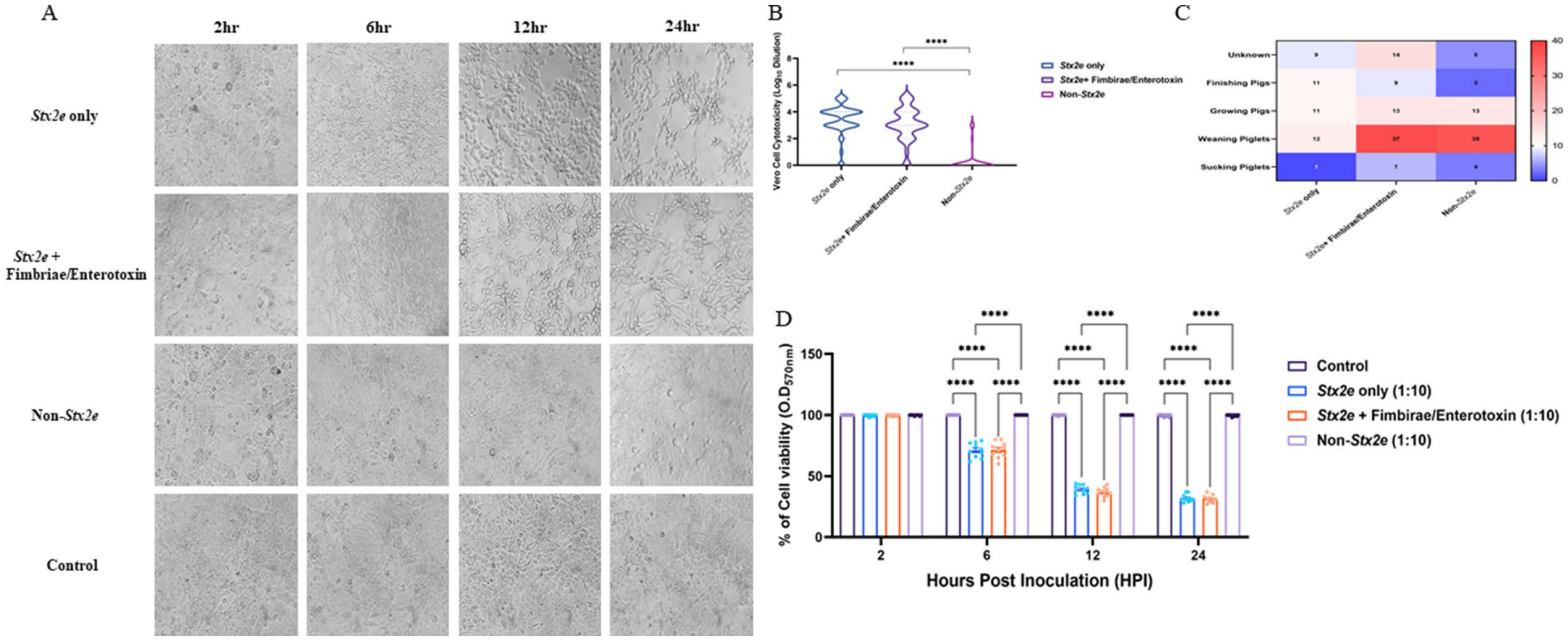
Cytotoxic effects of STEC isolates on Vero cells. **(A)** Morphological changes in Vero cells exposed to selected STEC isolates at 2-, 6-, 12-, and 24-hours post-exposure. Characteristic cytopathic effects including cell shrinkage and detachment were observed with earlier and more pronounced effects inisolates identified the stx2e gene compared to non-stx2e isolates. **(B)** Cytotoxic activity measured as the highest dilution factor causing 50% reduction in Vero cell viability (logio scale). Stx2e-positive groups demonstrated significantly greater cytotoxicity compared to the non-stx2e group (*p* < 0.0005). The stx2e-only group exhibited the highest median cytotoxic effect. **(C)** Distribution of STEC virulence groups across different pig production stages, showing predominance of stx2e-positive isolates at all ages. **(D)** Time-course analysis of cytotoxicity measured by MTT assay demonstrating significantly higher cytotoxic effects of stx2e-positive isolates compared to non-stx2e groups over 24 hours. Statistical significance was assessed using one-way ANOVA followed by Tukey's *post-hoc* test (*n*= 3 independent experiments per group). ****p< 0.0001.

Cytotoxic effects of selected STEC isolates were evaluated using a Vero cell-based MTT assay. Characteristic morphological alterations—such as cell shrinkage and detachment—were observed at 2-, 6-, 12-, and 24 h post-exposure (Figure 5A). Isolates containing the *stx2e* gene induced earlier and more pronounced cytopathic effects than the non-Stx2e group. Both Stx2e-positive groups, Stx2e-only, Stx2e + fimbriae/enterotoxin, exhibited significantly higher cytotoxic activity than the non-Stx2e group, as determined by the highest dilution factor resulting in a 50% reduction in cell viability (log_10_ scale). The Stx2e-only group showed a significantly (*p < 0.0005*) higher median cytotoxicity compared to the non-Stx2e group; however, the difference was not statistically significant compared to the Stx2e + Fimbriae/Enterotoxin group (Figure 5B). To investigate age-related patterns, a heatmap analysis was constructed to visualize the distribution of virulence groups across the pig production stages (Figure 5C). Quantitative absorbance data (OD_570_) from the MTT assay were also analysed to assess time-dependent cytotoxic responses across the three virulence categories (Figure 5D). These results complemented time-based cytotoxicity assessments and demonstrated that *Stx2e*-positive isolates showed significantly higher cytotoxicity compared to the non-Stx2e group. Stx2e-positive groups exhibited significantly higher cytotoxicity than non-Stx2e isolates (*p < 0.0005*), supporting that the assay reflects functional Stx2e activity relevant to vaccine strain evaluation rather than purified toxin characterization.

### 3.4 Comparative genomic and clonal structuring of representative STEC isolates

**Figure 6:**
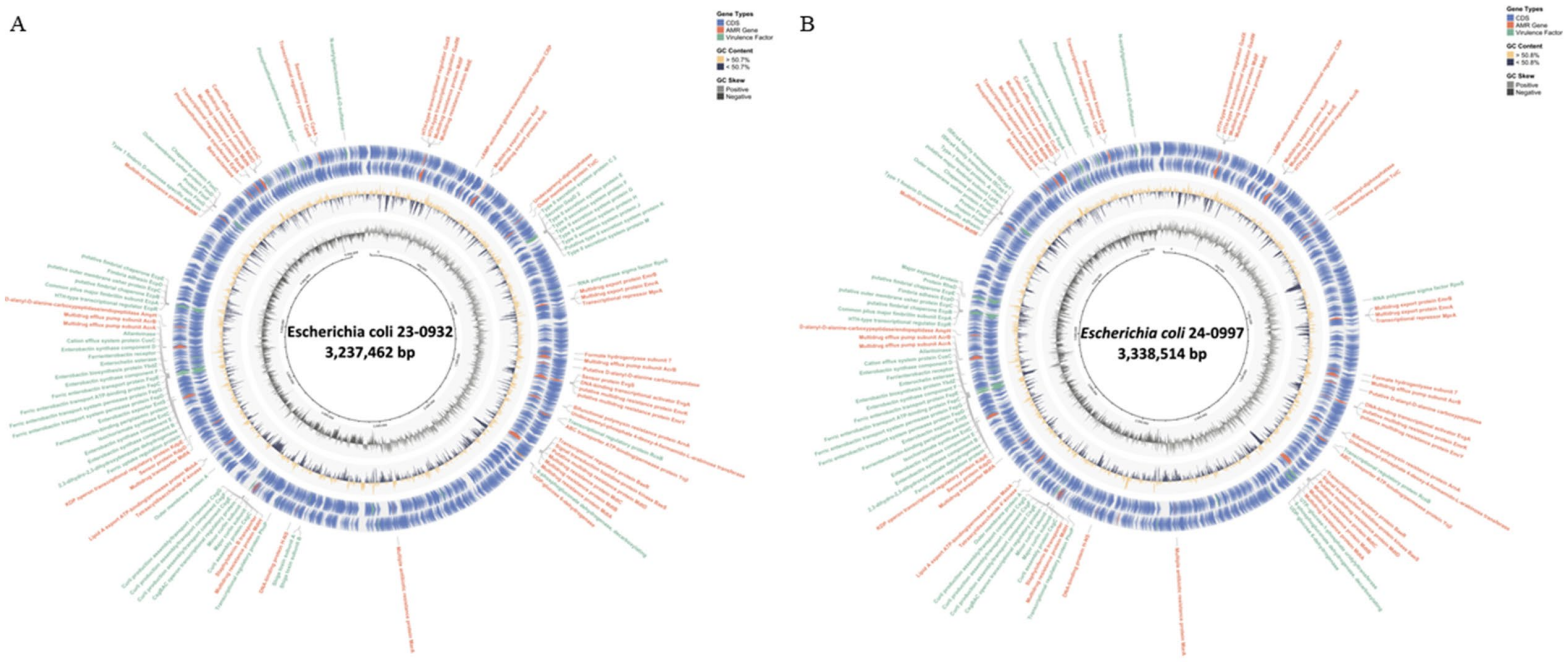
Circular genome visualization of two representative *Escherichia coli* isolates selected as vaccine candidates. **(A)** Genome map of *E. coli* 23–0932 (3,237,462,650 bp) showing core genes (CDS), virulence factors, antimicrobial resistance genes (AMR), GC content, and GC skew. **(B)** Genome map of *E. coli* 24–0997 (3,338,514,266 bp) with annotated virulence loci and mobile elements. The inner rings depict GC content (orange) and GC skew (positive in black, negative in grey), while the outermost annotations denote virulence-associated genes (green), AMR genes (red), and hypothetical proteins (blue). Gene classification was performed using Prokka and ABRicate pipelines and visualized using CGView.

Whole genome sequencing (WGS) was performed on two representative *E. coli* strains—23-0932 and 24-0997—selected based on their virulence gene profiles, toxin production levels, and *in vitro* growth characteristics. The circular genomic maps of both isolates were visualized to compare gene architecture, GC content, GC skew, and the distribution of virulence and antimicrobial resistance (AMR) genes (Figures 6A,B). Strain 23-0932 (genome size: 3,237,462 bp) displayed a compact core genome and clustered virulence-associated regions. Key virulence genes, including *stx2e, eae, and fimH*, were found adjacent to pathogenicity islands and flanked by mobile genetic elements. Notably, the *eae* gene is located within a partial Locus of Enterocyte Effacement (LEE) region, which is unusual for classical porcine Stx2e/ED isolates that are typically *eae–*. This arrangement may confer some potential for intimate adherence to intestinal epithelial cells, suggesting atypical pathogenic features in 23-0932. In-depth genomic context analysis confirmed that *eae* in strain 23-0932 is embedded within a partial but distinct LEE-like locus, flanked by mobile genetic elements. Although incomplete compared to canonical LEE islands observed in enteropathogenic *E. coli*, this architecture implies a potential for attaching and effacing (A/E)-like adherence. The presence of *eae* within such a genomic environment indicates that strain 23-0932 may represent an atypical evolutionary lineage of porcine Stx2e isolates, likely derived through horizontal gene transfer. This observation highlights the emergence of non-classical Stx2e/ED variants that may possess enhanced virulence plasticity or altered host-adaptation potential. Multiple antimicrobial resistance determinants, such as *tetA* and *sul1*, were detected in the outer ring annotations, suggesting horizontal gene transfer events. In contrast, strain 24-0997 (genome size: 3,338,514 bp) exhibited a more expansive genomic architecture, featuring multiple insertion sequences and transposase elements dispersed throughout the genome. Although both strains contained *stx2e*, their surrounding genomic context differed substantially, with 24-0997 exhibiting additional copies of *fimH* operons and an increased number of hypothetical proteins adjacent to known virulence loci. Both isolates showed balanced GC-skew distributions (innermost circle), with slight deviations near regions enriched in prophage-like elements. The overall GC content (outer second ring) was consistent with *E. coli* norms, averaging ~50.6%. Notably, the AMR gene content was more diverse in 23-0932, while 24-0997 carried multiple adhesin-related genes and unique secretion system components.

### 3.5 Multi-parameter selection of vaccine candidate strains

A total of 184 *E. coli* isolates were screened for the presence of the Stx2e gene to identify suitable vaccine candidate strains. Among these, Stx2e-positive isolates were prioritized due to their established role in edema disease pathogenesis. The Stx2e-only strains demonstrated significantly improved growth characteristics, including shorter lag phases, higher exponential growth rates, and increased maximum population densities, compared to strains harboring additional virulence factors. These findings indicate that Stx2e-only strains possess relatively high proliferation capacity, advantageous for large-scale vaccine antigen production, but also that Stx2e + fimbriae/enterotoxin strains exhibited high growth performance. In addition, the antimicrobial susceptibility testing revealed that a substantial proportion of Stx2e-only and Stx2e + fimbriae/enterotoxin strains exhibited multidrug resistance to most antibiotic classes. Therefore, to reflect the antimicrobial resistance profiles of circulating field strains, candidate strains displaying representative multidrug resistance profiles were selected to enhance vaccine relevance to circulating field strains. *In vitro* cytotoxicity assessed by the Vero cell MTT assay showed that both Stx2e-only and Stx2e + fimbriae/enterotoxin strains induced significantly higher cytotoxicity than non-Stx2e strains (Table 5).

**Table 5:** Integrated assessment to selected final vaccine candidates.

Vaccine candidate	WG-STEC-vaccine candidate 1 (23–0932)	WG-STEC-vaccine candidate 2 (24–0997)
Isolated specimens	Brain	Feces
Stx2e	+	+
Adhesion fimbriae (F4, F5, F18, F41)	F18	−
Enterotoxins (LT, STa, STb)	−	−
Growth kinetics (colony forming unit/mL)	4.0 × 10^8^ CFU/mL	4.2 × 10^8^ CFU/mL
Antibiotic classes in the MDR profile (abbreviations, *n*)	β-lactams (2), carbapenems (1), cephalosporin (4), aminoglycosides (3), quinolone (2), sulfonamide (2)	β-lactams (3), carbapenems (1), cephalosporin (5), aminoglycosides (3), macrolide (1), quinolone (2), tetracycline (1), sulfonamide (2)
Cytotoxicity (10-fold dilution)	High toxicity (10^5^) 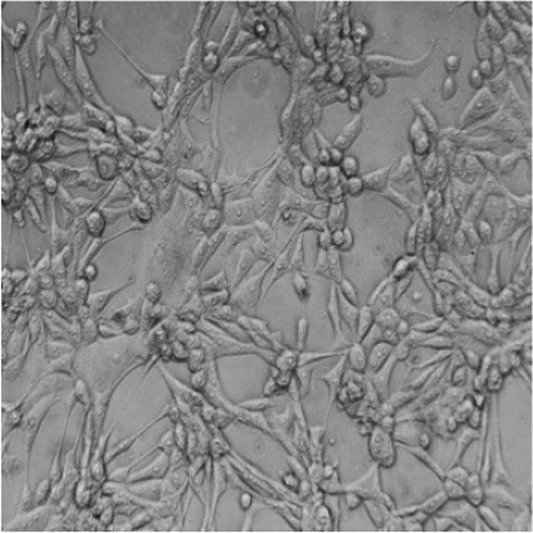	High toxicity (10^5^) 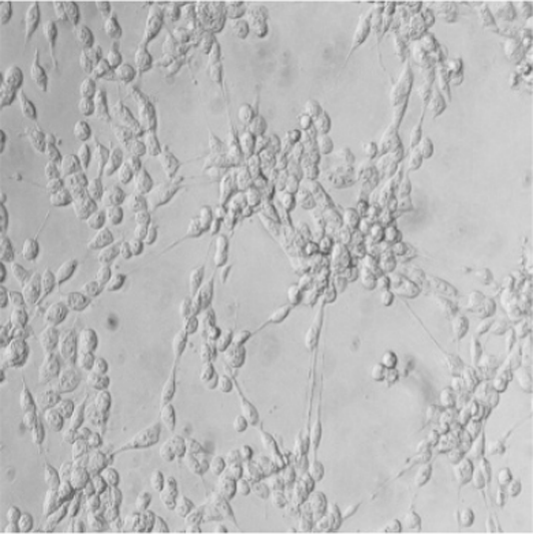

## 4 Discussion

Through the spatiotemporal (2014–2025) nationwide surveillance of Shiga toxin-producing *E. coli* (STEC), the diverse molecular epidemiology and characteristics were identified in the Republic of Korea. A total of 184 STEC isolates were characterized, with most recovered from gastrointestinal specimens, especially feces and intestinal tissues, reflecting a localized enteric infection pattern. This finding aligns with known clinical signs of STEC-related diseases in piglets, such as post-weaning diarrhea (PWD) and edema disease (ED), where the pathogen colonizes the small intestine and secretes potent toxins that induce systemic and local effects (Fairbrother et al., 2005). In addition, the weaning phases (5–8 weeks of age) of pig production stage were susceptible to STEC-infection complied with dietary changes, and an immature immune system as previously described (Madec et al., 2000). This emphasizes the urgent need for effective preventive strategies, including an appropriate time point of sows or suckling piglets, for vaccination.

In the genetic and molecular profiling of STEC isolates, 29 unique virulence gene combinations, including *stx2e*, F18 fimbriae, and enterotoxins such as LT, STa, and STb, were detected. Among them, the Stx2e and Stx2e *+* F18 genotypes were the most prevalent, indicating that these variants may be more frequently associated with edema diseases, colibacillosis, or other diseases (Baldo et al., 2020). Growth kinetics and PCA analyses based on toxin gene profiles revealed that Stx2e-only isolates exhibited relatively higher growth rates compared to other groups. This enhanced proliferative capacity was also distinguishable in the principal component space, suggesting a distinct growth kinetics associated with the absence of additional virulence gene factors. These findings may reflect a lower metabolic burden or altered regulatory dynamics in strains harboring stx2e-only, enabling more efficient proliferation under *in vitro* conditions (Kaper et al., 2004; Bai et al., 2016; Lim et al., 2010). This property could be considered favorable when selecting live-attenuated vaccine candidates, provided that virulence attenuation is concurrently validated.

The comparative whole-genome analysis of two representative *E. coli* isolates, 23-0932 and 24-0997, revealed notable genomic divergence despite sharing key virulence determinants, such as *stx2e*. The genomic organization patterns, presence of accessory gene clusters, and integration of mobile genetic elements indicated distinct evolutionary lineages, likely shaped by host-driven selective pressures and horizontal gene transfer (Zingali et al., 2020). Strain 23-0932 exhibited a more consolidated virulence profile, characterized by the presence of the *eae* gene located near pathogenicity-associated regions, although no complete LEE island was detected. This configuration suggests a potential for attaching and effacing–like interactions in porcine intestinal cells. In addition, the co-localization of antimicrobial resistance (AMR) and virulence determinants within genomic regions containing tRNA integration hotspots implies that horizontal gene transfer mediated by bacteriophages or plasmids may have contributed to this organization (Heo et al., 2020; Bai et al., 2016; Hacker and Kaper, 2000). Such genomic architecture supports the adaptive evolution of strain 23-0932 under antimicrobial selection pressures commonly encountered in pig production environments, while maintaining the Stx2e-dominant pathogenic mechanism characteristic of edema disease. In contrast, strain 24-0997 exhibited an expanded genomic structure, characterized by the presence of insertion sequences and unique adherence factors. This architectural complexity may reflect a broader metabolic or ecological niche, allowing the strain to persist under diverse gut microenvironments or environmental reservoirs (Kalalah et al., 2024). The presence of multiple adhesin systems and secretion-associated proteins also suggests a potential advantage in epithelial colonization, particularly under stress-induced gut permeability conditions common in weanling piglets. Interestingly, both strains maintained relatively conserved GC-skew patterns, supporting their genomic stability despite the insertion of foreign elements as previously described (Rocha, 2004). However, the distribution of hypothetical proteins adjacent to virulence loci in 24-0997 suggests unexplored functional diversity that may impact host-pathogen interaction or immune evasion mechanisms. Taken together, these results indicate that even within a single virulence genotype group (i.e., *stx2e*-positive STEC), significant genomic heterogeneity exists, which may influence vaccine design and efficacy (Brazzoli et al., 2023). The strain-specific genomic features identified here support the rationale for a multi-strain vaccine approach or the development of conserved antigen-based subunit vaccines targeting shared virulence determinants.

Antimicrobial susceptibility profiling of *Stx2e*-positive isolates revealed a high prevalence of multidrug resistance (MDR), with the majority of strains exhibiting resistance to at least three or more antimicrobial classes. Resistance was most frequently detected against tetracyclines, *β*-lactams (notably ampicillin), and sulfonamides—antimicrobial agents commonly used in swine production. This pattern aligns with international surveillance reports documenting similar resistance trends among porcine *E. coli* isolates, particularly those associated with post-weaning diarrhea and edema disease (Do et al., 2020; Ahmed and Shimamoto, 2015). The sustained exposure of field strains to selective antimicrobial pressure may have driven the accumulation of horizontally acquired resistance determinants, often located on mobile genetic elements such as plasmids and integrons (Ahmed and Shimamoto, 2015; Zhao et al., 2001). The near-complete absence of non-MDR isolates among *Stx2e*-harboring strains is particularly concerning, as it suggests that virulence and resistance traits are frequently co-localized, potentially amplifying clinical and epidemiological risks in affected herds. In the absence of effective countermeasures, the spread of MDR Stx2e strains could compromise treatment outcomes, exacerbate disease severity, and increase economic losses due to reduced growth performance and prolonged recovery periods (Mir and Kudva, 2019). In the veterinary field context, such multidrug resistance reflects cumulative selective pressure from farm-level antimicrobial use rather than therapeutic exposure alone (Daodu et al., 2025). These phenotypic profiles serve as epidemiological indicators for selecting representative vaccine seed strains under field conditions, emphasizing that the AMR analysis in this study was designed for vaccine development relevance rather than clinical MIC determination.

In Vero cell-based cytotoxicity assays, Stx2e-positive isolates consistently induced pronounced morphological changes and significant reductions in cell viability compared to non-Stx2e strains. The *Stx2e*-only group exhibited the most severe effects at early time points. Importantly, all isolates tested for cytotoxicity were previously confirmed to carry the stx2e gene by PCR, supporting the interpretation that observed effects predominantly reflect Stx2e activity. We acknowledge that Vero cell assays are not fully Stx2e-specific, and future studies are planned to include orthogonal confirmation, such as neutralization with anti-Stx2e antibodies and Gb4/Gb5 receptor competition assays. Notably, a subset of Stx2e-only isolates maintained strong cytotoxic potential despite lacking F18 fimbriae, suggesting the involvement of alternative virulence mechanisms capable of mediating host cell damage. These results highlight *Stx2e* as a key functional immunogen, independent of traditional colonization factors, and support its inclusion in vaccine antigen design (Tozzoli et al., 2014). In veterinary vaccinology, such functional correlation between *stx2e* genotype and Vero cytotoxicity provides a practical and ethically sustainable criterion for field strain selection before confirmatory toxin-neutralization or receptor-binding assays are undertaken in subsequent antigen validation studies.

Currently available vaccines for edema disease in swine largely target F18-positive strains, primarily through fimbrial or toxoid-based formulations. However, field reports increasingly describe the emergence of *Stx2e*-producing STEC isolates that lack F18 or harbor divergent virulence gene profiles, often correlating with suboptimal vaccine efficacy or clinical outbreaks in immunized herds (Baldo et al., 2020). Such strains have been recovered across all production stages, suggesting an expanding antigenic diversity that is inadequately addressed by existing vaccine platforms (Kim et al., 2010; Makino et al., 2001). This underscores the urgent need for broadened immunogen selection strategies that prioritize conserved toxins such as *Stx2e* lineage-specific colonization antigens, especially in regions with high genetic variability among circulating STEC isolates (Baranzoni et al., 2016; Galarce et al., 2019).

To address this, an integrated evaluation framework was applied to identify candidate vaccine strains that reflect both field relevance and production feasibility. Two representative *Stx2e*-positive isolates were selected based on a composite scoring system incorporating virulence profile, *in vitro* growth performance, cytotoxicity, and antimicrobial resistance. These strains demonstrated strong and stable proliferation (>4.0 × 10^8^ CFU/mL), high cytotoxicity (10^5^ titer), and multidrug resistance patterns consistent with those observed in the broader strain collection. One of the selected isolates carried the F18 fimbrial gene, while the other did not, thereby providing complementary antigenic coverage. This dual-candidate approach ensures a more comprehensive immunological response while maintaining manufacturing compatibility, offering a rational and scalable strategy for next-generation vaccine development against edema disease in swine. Furthermore, both candidate strains exhibited consistent in vitro phenotypic profiles across multiple passages, indicating preliminary stability at the functional level. This phenotypic reproducibility complements the genomic evidence of stability derived from WGS analyses. To ensure long-term reliability of the vaccine seed strains, serial passage and *in vivo* stability testing will be conducted in subsequent development stages. Based on these results, efforts are currently underway to formulate and evaluate multiple vaccine platforms, including toxoid-based, recombinant subunit, exosome-based, and inactivated vaccine formulations, leveraging the antigenic potential of selected strains to provide robust protection against a broader spectrum of field-circulating STEC. This approach, supported by integration of molecular, phenotypic, virotype, and epidemiological results, enabled the selection of vaccine candidates that not only exhibit strong immunological potential but also align with the practical demands of large-scale production and implementation. Future work will focus on pilot-scale vaccine development, in vivo efficacy testing, and field application trials to validate safety, immunogenicity, and impact on disease prevalence and antimicrobial usage in swine farms.

## 5 Conclusion

Through a nationwide molecular epidemiological surveillance spanning 2014 to 2025, this study characterized the virulence gene diversity, antimicrobial resistance, and in vitro phenotypes of porcine Shiga toxin-producing *Escherichia coli* (STEC) isolates circulating in Korea. The predominance of Stx2e-positive strains, coupled with the detection of highly heterogeneous virulence gene constellations—including both F18-positive and F18-negative genotypes—highlights the evolving antigenic landscape of field strains and the challenges it poses to current vaccine strategies. The widespread occurrence of multidrug resistance (MDR) among toxin-containing isolates further emphasizes the urgency of developing effective non-antibiotic interventions. By integrating genotypic and phenotypic datasets, two representative Stx2e-positive isolates were selected as rational vaccine candidates based on their strong cytotoxicity, high in vitro proliferation capacity, cell cytotoxicity, and relevant antimicrobial resistance profiles. Notably, the evaluation of both F18-positive and F18-negative isolates allowed for the identification of a vaccine candidate with broader antigenic representativeness, addressing the limitations of current vaccine formulations. This selection strategy, supported by a nationwide longitudinal surveillance dataset spanning 2014–2025, offers a rational and evidence-based foundation for the development of next-generation immunogens against porcine edema disease. The selected candidate strains are currently under evaluation across multiple vaccine platforms, including toxoid-based, recombinant subunit, exosome-derived, and inactivated vaccine formulations, designed to align with the production parameters and epidemiological landscape of the Korean swine industry. This strategy constitutes a data-driven framework for non-antibiotic disease control, aiming to reduce STEC-associated clinical and economic burdens through immunologically relevant and manufacturing-compatible vaccine solutions.

## Data Availability

The raw data supporting the conclusions of this article are included in the article and its supplementary material. Additional data will be made available by the authors upon reasonable request.
