# Supplementary material for: Phenotypic and genotypic profiling of swine-derived Shiga toxin-producing Escherichia coli over a decade in South Korea: a framework for edema disease vaccine candidate strains selection
Source: Front Microbiol. 2025 Dec 16;16:1701708. doi: 10.3389/fmicb.2025.1701708 (PMC12748232; doi:10.3389/fmicb.2025.1701708)
Supplement: Supplementary file 1 [file Data_Sheet_1.docx]

**Supplementary Table 1. Gene Sequence Similarity and Divergence of Representative STEC Vaccine Candidate Strains**

| **No.** | **Strain name** | **% Similarity with strain no.** | | | | | | | | | | | | | | | | | | | | | | | |
| --- | --- | --- | --- | --- | --- | --- | --- | --- | --- | --- | --- | --- | --- | --- | --- | --- | --- | --- | --- | --- | --- | --- | --- | --- | --- |
|  |  | 1 | 2 | 3 | 4 | 5 | 6 | 7 | 8 | 9 | 10 | 11 | 12 | 13 | 14 | 15 | 16 | 17 | 18 | 19 | 20 | 21 | 22 | 23 | 24 |
| 1 | *E. coli* 23-0932 | - | 100 | 100 | 100 | 100 | 100 | 100 | 100 | 100 | 100 | 100 | 82.1 | 97.2 | 97.3 | 97.3 | 97.3 | 97.3 | 97.3 | 97.3 | 97.3 | 97.3 | 97.3 | 97.3 | 82.5 |
| 2 | *E. coli* t2.2 | 2.8 | - | 100 | 89.1 | 100 | 96.2 | 87.9 | 87.6 | 89.1 | 87.9 | 87 | 78.8 | 97.2 | 89 | 97.3 | 96.2 | 96.2 | 96.2 | 96.2 | 96.2 | 96.2 | 96.2 | 96.2 | 79.2 |
| 3 | *E. fergusonii* C6-A-0-1-65 | 2.8 | 0 | - | 97.3 | 100 | 97.3 | 91.2 | 91.1 | 92.5 | 92 | 90.8 | 81.9 | 97.2 | 97.3 | 97.3 | 96.2 | 96.2 | 96.2 | 96.2 | 96.2 | 96.2 | 96.2 | 96.2 | 82.3 |
| 4 | *E. fergusonii* | 2 | 1.9 | 1.9 | - | 100 | 100 | 90.1 | 89.4 | 89.8 | 88.8 | 89.1 | 78.6 | 97.2 | 88.8 | 97.3 | 96.2 | 95.4 | 96.2 | 96.2 | 96.2 | 96.2 | 96.2 | 96.2 | 78.9 |
| 5 | *E. fergusonii* MH-B1 | 2.8 | 0 | 0 | 1.9 | - | 100 | 92.9 | 92.4 | 93.4 | 92.9 | 91.5 | 82.4 | 97.2 | 97.3 | 97.3 | 96.2 | 96.2 | 96.2 | 96.2 | 96.2 | 96.2 | 96.2 | 96.2 | 82.8 |
| 6 | *E. coli* AUMC B-521 | 2 | 1.9 | 1.9 | 0 | 1.9 | - | 90.8 | 91.2 | 92.5 | 92 | 90.8 | 81.7 | 97.2 | 95.4 | 97.3 | 96.2 | 95.4 | 96.2 | 96.2 | 96.2 | 96.2 | 96.2 | 96.2 | 82.1 |
| 7 | *Shigella sp*. CH-43 | 0.3 | 3.1 | 3.1 | 2.3 | 3.1 | 2.3 | - | 94.7 | 92.5 | 93.5 | 93 | 77.7 | 97.2 | 87.3 | 92.9 | 92.4 | 90.4 | 91.1 | 91.1 | 91.6 | 91.6 | 91.6 | 91.1 | 78 |
| 8 | *Shigella sp.* CH-41 | 0.3 | 3.1 | 3.1 | 2.3 | 3.1 | 2.3 | 0.6 | - | 90.8 | 92.1 | 91.6 | 77.7 | 97.2 | 87.3 | 92.4 | 92 | 90.4 | 90.8 | 90.7 | 91.1 | 91.1 | 91.1 | 90.7 | 78 |
| 9 | *Shigella sp.* CH-40 | 1 | 2.4 | 2.4 | 1.6 | 2.4 | 1.6 | 1.2 | 1.3 | - | 89.1 | 89.4 | 78.4 | 97.2 | 88.5 | 93.4 | 92.9 | 91.6 | 91.6 | 91.5 | 92 | 92 | 92 | 91.6 | 78.7 |
| 10 | *Shigella sp.* CH-33 | 0.7 | 3.5 | 3.5 | 2.7 | 3.5 | 2.7 | 0.7 | 1 | 1.7 | - | 91.2 | 77.6 | 97.2 | 87 | 92.9 | 92.4 | 91.1 | 91.1 | 91.1 | 91.6 | 91.6 | 91.6 | 91.1 | 77.9 |
| 11 | Shigella sp. CH-43 | 0.5 | 3.3 | 3.3 | 2.5 | 3.3 | 2.5 | 0.8 | 0.8 | 1.5 | 1.2 | - | 77.3 | 97.2 | 86.5 | 91.5 | 91.1 | 90 | 90 | 90 | 90.4 | 90.4 | 90.4 | 90 | 77.6 |
| 12 | *S. enterica* LT2 | 6.6 | 5.1 | 5.1 | 5.6 | 5.1 | 5.6 | 6.8 | 6.9 | 6.2 | 7.3 | 7.1 | - | 81.5 | 82.2 | 82.3 | 82 | 81.9 | 81.9 | 82.1 | 81.9 | 81.9 | 81.9 | 82.1 | 91.9 |
| 13 | *E. coli* 24-0997 | 3 | 1.5 | 1.5 | 2.1 | 1.5 | 2.1 | 3.3 | 3.3 | 2.6 | 3.7 | 3.5 | 3.8 | - | 100 | 100 | 100 | 100 | 100 | 100 | 100 | 100 | 100 | 100 | 82.2 |
| 14 | *S. sonnei* SE6-1 | 2.9 | 1.4 | 1.4 | 2 | 1.4 | 2 | 3.2 | 3.3 | 2.5 | 3.7 | 3.5 | 3.7 | 0.1 | - | 100 | 97.3 | 97.3 | 97.3 | 97.3 | 97.3 | 97.3 | 97.3 | 97.3 | 82.5 |
| 15 | *E. coli* NF73_8 | 2.9 | 1.4 | 1.4 | 2 | 1.4 | 2 | 3.2 | 3.3 | 2.5 | 3.7 | 3.5 | 3.7 | 0.1 | 0 | - | 97.3 | 97.3 | 97.3 | 97.3 | 97.3 | 97.3 | 97.3 | 97.3 | 82.7 |
| 16 | *E. coli* NF73_7 | 3 | 1.5 | 1.5 | 2.1 | 1.5 | 2.1 | 3.3 | 3.3 | 2.6 | 3.7 | 3.5 | 3.8 | 0 | 0.1 | 0.1 | - | 100 | 100 | 100 | 100 | 100 | 100 | 100 | 82.4 |
| 17 | *E. coli* NF73_73 | 3 | 1.5 | 1.5 | 2.1 | 1.5 | 2.1 | 3.3 | 3.3 | 2.6 | 3.7 | 3.5 | 3.8 | 0 | 0.1 | 0.1 | 0 | - | 100 | 100 | 100 | 100 | 100 | 100 | 82.3 |
| 18 | *E. coli* NF73_72 | 3 | 1.5 | 1.5 | 2.1 | 1.5 | 2.1 | 3.3 | 3.3 | 2.6 | 3.7 | 3.5 | 3.8 | 0 | 0.1 | 0.1 | 0 | 0 | - | 100 | 100 | 100 | 100 | 100 | 82.3 |
| 19 | *E. coli* NF73_71 | 3 | 1.5 | 1.5 | 2.1 | 1.5 | 2.1 | 3.3 | 3.3 | 2.6 | 3.7 | 3.5 | 3.8 | 0 | 0.1 | 0.1 | 0 | 0 | 0 | - | 100 | 100 | 100 | 100 | 82.5 |
| 20 | *E. coli* NF73_70 | 3 | 1.5 | 1.5 | 2.1 | 1.5 | 2.1 | 3.3 | 3.3 | 2.6 | 3.7 | 3.5 | 3.8 | 0 | 0.1 | 0.1 | 0 | 0 | 0 | 0 | - | 100 | 100 | 100 | 82.3 |
| 21 | *E. coli* NF73_6 | 3 | 1.5 | 1.5 | 2.1 | 1.5 | 2.1 | 3.3 | 3.3 | 2.6 | 3.7 | 3.5 | 3.8 | 0 | 0.1 | 0.1 | 0 | 0 | 0 | 0 | 0 | - | 100 | 100 | 82.3 |
| 22 | *E. coli* NF73_69 | 3 | 1.5 | 1.5 | 2.1 | 1.5 | 2.1 | 3.3 | 3.3 | 2.6 | 3.7 | 3.5 | 3.8 | 0 | 0.1 | 0.1 | 0 | 0 | 0 | 0 | 0 | 0 | - | 100 | 82.3 |
| 23 | *E. coli* NF73_67 | 3 | 1.5 | 1.5 | 2.1 | 1.5 | 2.1 | 3.3 | 3.3 | 2.6 | 3.7 | 3.5 | 3.8 | 0 | 0.1 | 0.1 | 0 | 0 | 0 | 0 | 0 | 0 | 0 | - | 82.5 |
| 24 | *S. enterica* Ty2 | 6.4 | 4.9 | 4.9 | 5.5 | 4.9 | 5.5 | 6.7 | 6.7 | 6 | 7.1 | 6.9 | 0.7 | 3.6 | 3.6 | 3.6 | 3.6 | 3.6 | 3.6 | 3.6 | 3.6 | 3.6 | 3.6 | 3.6 | - |

16s rRNA gene sequence similarity and divergence
