# Supplementary figures and images for: Phenotypic and genotypic profiling of swine-derived Shiga toxin-producing Escherichia coli over a decade in South Korea: a framework for edema disease vaccine candidate strains selection
Source: Front Microbiol. 2025 Dec 16;16:1701708. doi: 10.3389/fmicb.2025.1701708 (PMC12748232; doi:10.3389/fmicb.2025.1701708)

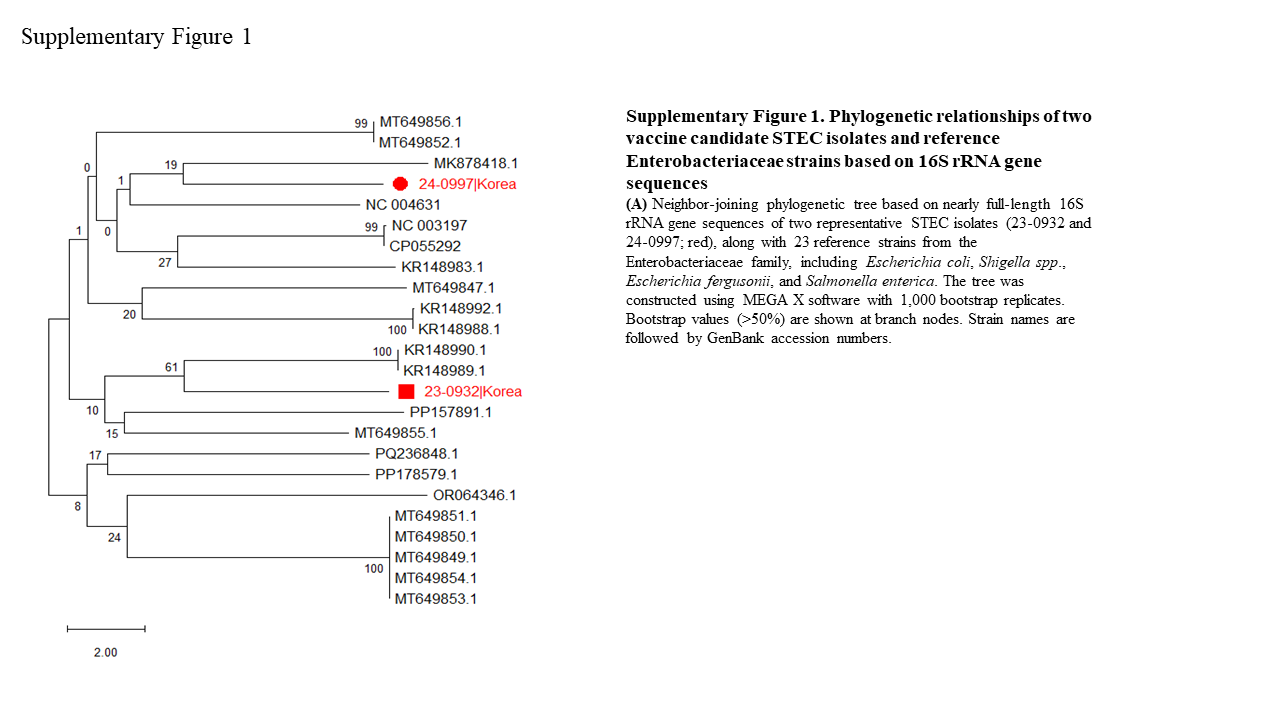

Supplement: Supplementary file 2 [file Image_1.tif]
